# Ameloblastic Carcinoma: A 40-Year Scoping Review of the Literature

**DOI:** 10.3390/cimb47040261

**Published:** 2025-04-08

**Authors:** Maria Giulia Cristofaro, Ida Barca, Angelo R. Sottile, Francesco Ferragina

**Affiliations:** Maxillo-Facial Surgery Unit, Department of Experimental and Clinical Medicine, “Magna Graecia” University, Viale Europa, 88100 Catanzaro, Italy; barca.ida@gmail.com (I.B.); angelo.sottile.1991@gmail.com (A.R.S.); francesco.ferragina92@gmail.com (F.F.)

**Keywords:** maxillofacial surgery, odontogenic tumors, head and neck cancer, oral health, ameloblastic carcinoma

## Abstract

Background: Ameloblastic carcinoma (AC) is a rare malignant odontogenic tumor with limited knowledge surrounding its pathogenesis, molecular pathways, clinical behavior, treatment, and prognosis. This 40-year literature scoping review aims to enhance the comprehension of this complex condition, looking closely at how AC works at molecular and pathophysiological levels and what causes it to develop. Methods: The PUBMED, Medline, Scopus, and Cochrane central databases were searched, including articles from 1984 to date. Articles reporting epidemiological, clinical, instrumental, and histopathological data were included. Results: Out of the 375 articles examined, 52 met the inclusion criteria, yielding a total of 80 cases of AC. All cases before 1984 were excluded from the analysis, as were all that did not provide information on patient survival. Several molecular mechanisms associated with its development and progression were identified; these help in early diagnosis. Moreover, AC can spread locally, making a radical surgical approach necessary. There is still no agreement on how to manage neck dissection. Surgical removal followed by monitoring is an important part of managing AC. Conclusions: Advancements in biological and molecular insights have the potential to facilitate earlier diagnosis and treatment. These could lead to improvements in patients’ quality of life and long-term survival.

## 1. Introduction

Odontogenic tumors represent a heterogeneous group of rare lesions, comprising less than 5% of all tumors [1]. These cells are derived from epithelial and mesenchymal tissues and are located almost exclusively in the maxillary bones. The etiology of these lesions remains unknown. In most cases, these lesions are benign, with less than 10% exhibiting malignant characteristics [1,2,3].

In 1982, Elzay first defined AC as a tumor with histological characteristics of ameloblastoma and concomitant squamous cell carcinoma.

In 1984, Slootweg and Muller distinguished metastatic ameloblastoma into two principal forms: malignant ameloblastoma (MA) and AC [4]. Both entities are collectively designated as primary intraosseous carcinoma ex ameloblastoma. The classification of malignant tumors is based on differentiation. The categories include the following:

Type 1: Primary intraosseous carcinoma ex odontogenic cyst.

Type 2: Primary intraosseous carcinoma ex ameloblastoma,

- Malignant ameloblastoma.
- Ameloblastic carcinoma (occurs de novo, ex ameloblastoma or ex odontogenic cyst).

Type 3: Primary intraosseous carcinoma that occurs de novo.

- Non-keratinizing.
- Keratinizing.

After decades of controversy, the World Health Organization (WHO) classified the malignant counterpart of ameloblastoma into two distinct types: malignant ameloblastoma (MA) and ameloblastic carcinoma (AC). MA exhibits histological similarities to benign tumors but demonstrates the capacity for long-range metastatic spread. AC can be further classified into two subtypes, primary and secondary forms. Primary AC develops de novo, whereas secondary AC (also referred to as intraosseous or peripheral) arises as an evolution of a previously diagnosed benign ameloblastoma.

In 2017, the WHO proposed a reclassification of odontogenic tumors, removing the sub-classifications that had previously been in place. In this manner, WHO sought to eliminate diagnostic ambiguity. The new classification was based on the site of tumor development and diagnostic criteria. Furthermore, the differentiation between primary and secondary AC has been rendered moot.

In 2022, the WHO presented the fifth edition of the classification, which, in conceptual terms, is not significantly different from that of the 2017 classification [5]. AC is regarded as a primary odontogenic carcinoma with a histological profile analogous to ameloblastoma. BRAF p.V600E mutations, the most common activating mutation in conventional ameloblastoma, have been identified in AC, but their diagnostic value remains uncertain.

The paucity of the literature on AC limits is evidenced by the predominance of case reports and series of small cases. This suggests a paucity of clear and unambiguous guidelines regarding its management and treatment. Moreover, substantial variations in clinical practice exist among countries, as well as within their respective health services, models of care, and access to care.

To address this knowledge gap, a comprehensive review was conducted to systematically map the extant research in this area and identify knowledge gaps. This review encompasses 40 years of literature on CA cases from 1984 to the present. This scoping review is a valuable addition to the existing body of knowledge in the CA literature, offering crucial insights into the clinical and radiological characteristics, treatment modalities, recurrence rates, and survival outcomes associated with this malignancy.

## 2. Methods

**Figure 1:**
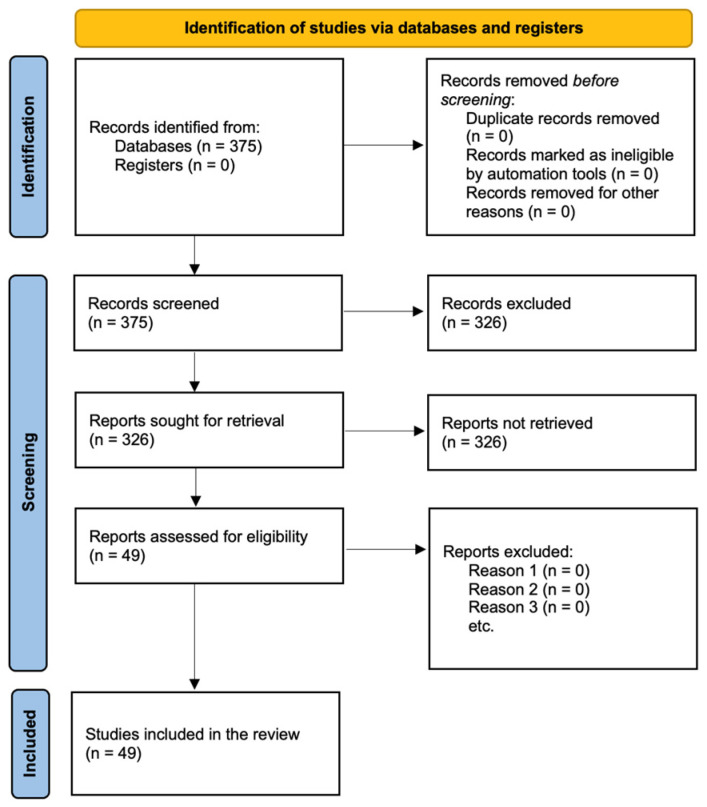
Preferred Reporting Items for Systematic Review and Meta-Analysis (PRISMA) flowchart for the searching and identification of included studies. (n = number of studies).

A 40-year scoping review of the literature was conducted following the Preferred Reporting Items for Systematic Reviews and Meta-Analyses (PRISMA) Statement guidelines (Figure 1) [6]. The study protocol was registered in the PROSPERO database under the identification number 517490.

### 2.1. Search Strategy

In November 2024, a comprehensive search was conducted in the following electronic databases: Scopus (Elsevier), Web of Science (Clarivate Analytics), PubMed (National Library of Medicine), Medline, and Cochrane Central databases. The keyword “ameloblastic carcinoma” was used in all searches, and the “related articles” function was used to expand the scope of the search. Following a preliminary review of the subject matter, a research strategy was devised utilizing the following keywords: ameloblastic carcinoma, ameloblastoma, malignant ameloblastoma, and odontogenic tumors. Two authors (FF and IB) conducted independent screenings and identified the articles for inclusion, consulting a third senior author (MGC) in cases of significant discrepancies.

### 2.2. Inclusion Criteria and Study Selection

The inclusion criteria were applied during title, abstract, and full-text screening following the PICO framework.

(1) P: patients diagnosed with ameloblastic carcinoma from 1984 to 2024.
(2) I: patients who underwent standard treatments, such as surgery or adjuvant treatments.
(3) C: patients who did not undergo any type of treatment.
(4) O: studies that reported outcomes and/or complications of ameloblastic carcinoma with a minimum follow-up of six months.

Other articles that did not present outcomes or results, technical notes, editorials, letters to the editor, or expert opinions were excluded from the analysis but were considered for inclusion in the discussion section.

### 2.3. Data Extraction and Quality Assessment

Two maxillofacial surgeons (FF and IB) conducted the initial examination of the included studies and extracted the pertinent data. For each article, the following information was recorded: the first author, the journal name, the year of publication, the scientific level, the type of surgery, and the patient demographics.

The data extracted for quantitative analysis were limited to cases of surgical or standard adjuvant carcinoma. The titles and abstracts were read and evaluated, and if a study was deemed potentially relevant, the full text of the publication was reviewed. Upon the completion of the comprehensive review of the literature, the following data were extracted: characteristics of the study (the author, publication year, and study design), patient demographics, and clinical data (tumor location, diagnosis, histological data, treatment, possible metastases, recurrence, survival, complications, and follow-up).

The data were subjected to an aggregate examination to create a cumulative review. All data were collected and tabulated using a Microsoft Excel spreadsheet. The two authors conducted an independent assessment of the methodological quality. The risk of bias tool, version 2, was used to assess randomized controlled trials in accordance with the recommendations of the Cochrane Collaboration. Any discrepancies were resolved through consultation with a senior reviewer with over 15 years of experience in maxillofacial surgery (MGC).

## 3. Results

A comprehensive review of the literature was conducted, encompassing all 289 cases of AC from 1927 to 2024. All cases preceding 1984 were excluded from the analysis, as were all cases that did not provide information regarding patient survival. A five-year cut-off for survival was employed, with patients divided into two groups: those who survived more than five years (Table 1) and those who survived less than five years (Table 2).

The mean age of the subjects was 46.93 years, with an age range of 2 to 90 years. Of all the studied subjects, 29 (29%) were women, and 71 (71%) were men (yielding a male-to-female ratio of 2.45:1). A total of 66 cases of AC were identified in the mandible, with 33 cases observed in the maxilla. This resulted in a mandible-to-maxilla ratio of 2:1. Eight cases (10%) exhibited evidence of metastasis to the lymph nodes, with many cases specifying that they were cervical lymph nodes and others identifying them as locoregional. In 29 cases (36.25%), distant metastases were identified. Of these, twelve (15%) were in the lung, one (1.25%) in the kidney, three (3.75%) in the liver, six cases (7.5%) in the bones (three in the skull, one in the spine, and two in the other bones), three (3.75%) cases in the brain, one (1.25%) in the pterygomaxillary fossa, and three (3.75%) in the orbit.

AC with a mandibular origin demonstrated a 26.41% propensity for distant metastasis, with the lungs representing the most frequent site (20.75%), followed by locoregional lymph nodes (9.43%). AC with maxilla origin metastasized in 23.08% of cases, with the most frequent site being the lung and the next most frequent being the locoregional lymph nodes. AC with skull base origin metastasized in 33.33% of cases, and one in three cases revealed intracranial metastases.

The duration of the disease is highly variable and contingent upon the specific type and timing of therapy. A systematic review proposed by Giridhar et al. (199 cases of AC) revealed the following distribution of treatment: 56.3% of cases involved surgery alone, 31.2% involved surgery followed by radiotherapy, 6.5% involved radiotherapy alone, and 6.0% involved no treatment or palliative care. Patients with localized disease, younger age, and those who underwent R0 resection had favorable survival. Radiation therapy did not confer a survival benefit, except in elderly patients with multiple comorbidities and who were therefore ineligible for surgery, who have may benefitted [55].

Regrettably, no objective data on survival have emerged. Most of the literature presents data in the form of case reports, which do not provide objective and standardized data on this aspect. Therefore, it is not feasible to ascertain the survival rate of this malignant tumor with precision.

### 3.1. Epidemiology

AC is more common in males, with a sex ratio M:F of 2.03:1. It mainly affects the adult population with an average age of 48.63 years. A correlation between sex and age of onset was observed, with the average age of onset in males being 53.70 years and 37.68 years in females. This indicates that, while AC is more prevalent among males, it manifests at an earlier age in females. The most common site is the mandible, with a mandible–maxilla ratio of 2.04:1; only two cases from the base of the skull and one case from the neck are reported in the literature. The posterior mandible is the most common site, followed by the anterior mandible and maxilla [55]. The exact demographics of AC are not clear as they are more described in case reports; the most impacted country appears to be the United States of America [56], which can be a sign of increased sensitivity to diagnosis rather than a real increase in the incidence of the disease itself.

### 3.2. Clinical Features

The clinical presentation is heterogeneous. The most common onset symptoms were swelling (62%), discomfort (48%), pain (37%), and numbness (24%). The less common symptoms included tooth loss (15%), gingival ulcers (12%), nasal obstruction/congestion (10%), gingival suppurating (3%), hemorrhage (3%), and lockjaw (2%).

AC may result in locoregional involvement or produce regional and distant metastases; these lesions metastasize mainly to the lung or regional lymph nodes [42]. Clinically, this pulmonary involvement may present with cough, dyspnea, hemoptysis, and, rarely, a paraneoplastic syndrome. These pulmonary metastases are often indolent clinically and have a long median survival. Lymph nodes are the second most common site of metastasis, while other rare sites include the brain, bone marrow, and liver [16,49,57,58].

### 3.3. Radiologic Features

For diagnosis, panoramic orthography is used as a first-line investigation; computed tomography (CT) and magnetic resonance imaging (MRI) are important second-line investigations. On radiographs, AC appears as a radiolucent/translucent multilocular lesion, the margins of which are usually poorly defined (there is an invasion of the surrounding bone). It has a nuanced appearance defined as a “honeycomb” or “bubble”. The presence of some focal radiopacities reflecting dystrophic calcifications may help in the differential diagnosis of ameloblastoma. The teeth may be affected by the resorption of the roots. Corio et al. in 1987 listed several radiological features of AC that are also found in the desmoplastic variant of conventional ameloblastoma [59]. However, these are not specific to AC. This wide variety of radiological features makes the interpretation of instrumental images (conventional radiography, CT, and MRI) difficult.

MRI is the diagnostic gold standard for differentiating between the various AC models [12,50,56]. Solid components tend to have a non-homogeneous intermediate signal on T1-weighted images and a non-homogeneous high signal on T2-weighted images and after contrast administration. Cystic components tend to have a homogeneous intermediate signal on T1-weighted images and a homogeneous high signal on T2-weighted images. The latter do not show a high signal after contrast administration.

CT, on the other hand, shows a radiolucent intraosseous mass with varying degrees of dystrophic calcification; post-contrast may also show the extensive involvement of surrounding soft tissues [50].

In this study, 42.89% of AC cases appear as osteolytic destruction and dystrophic calcification on CT; in 2.09% of maxillary AC cases, the bone cortex is absorbed with the subsequent invasion of the nasal cavity.

### 3.4. Histological and Immunohistochemical Features

AC is defined by a combination of cytological features of malignancy and a histological model of ameloblastoma, both in primary and metastatic lesions [25,51,54]. Its architecture is very variable; it can be arranged in follicular or plexiform grids (typical of ameloblastoma) or organized in sheets, nests, or trabeculae of epithelium. The most common form is the follicular reticulum. Peripheral cells show a typical palisade organization and invert nuclear polarity [25,54]. Central cells are densely arranged and may show typical hypercellular areas organized in a star pattern (present in only 16.7% of cases) [24]. The normal cellular organization of the tissue is disturbed, resulting in the following: the loss of stratification between basal and intermediate layers, pleomorphism, increased nucleus (nuclear hyperchromatism), increased mitotic activity, abnormal mitosis, vascular and/or perineural invasion, and necrosis. Necrosis is rare and is always considered a sign of malignancy. Dystrophic calcifications and clear cells may be present in 3% of cases. A morphology like that of the primary tumor is seen in the histological analysis of lymph nodes and distant metastases.

Because of this variability, histology may be inconclusive. Immunohistochemical analysis, therefore, allows us to diagnose AC with certainty.

Yoon et al. compared several immunohistochemical markers expressed by ameloblastoma and AC. Cytokeratin 18, matrix metalloproteinase-2 (MMP-2), stromal matrix metalloproteinase-9 (MMP-9), and Ki-67 were shown to discriminate between the two entities. The high levels of these proteins indicate high proliferative activity and allow the diagnosis of AC to be made concerning benign ameloblastoma. This study showed significant positivity, especially for cytokeratin 18 and Ki-67 (mean of 16.31% with an interval of 9.30–22.9%). Positive reactivity for p53 was also found in 75% of tumor cells. In addition, immunoreactivity for vimentin, desmin, neuron-specific enolases, common leukocyte antigen, CD99, and S-100 proteins was negative in that study [60].

Lei et al. attempted to make a differential diagnosis between these two entities by looking for SOX2, a protein involved in the ameloblastic epithelial lineage. SOX2 showed a diffuse and strong nuclear staining model with a specificity of 86.4% and a sensitivity of 76.9% in AC compared to the benign counterpart [61].

The molecular analysis used by Khojasteh et al. showed a statistically significant presence of the cyclin-dependent 2A kinase inhibitor mutation p16 in all AC samples compared to ameloblastoma, suggesting that the alteration of p16 may play a role in the malignant progression of AC [62].

All this underlines the importance of microscopic observation and adequate sampling, especially immunohistochemical analysis, in diagnosing AC.

### 3.5. Molecular Mechanisms and Pathophysiology

Several molecular mechanisms associated with developing and progressing AC have been identified. The alterations in the Wnt/β-catenin pathway have been associated with more advanced/aggressive disease in several tumors. β-Catenin (encoded by CTNNB1) is an important component of intercellular junctions, such as the components of adherens junctions, and its aberrant expression has been associated with many malignant neoplasms. The Wnt/β-Catenin signaling pathway plays a critical role in regulating various cellular processes, including proliferation, differentiation, survival, and epithelial–mesenchymal interactions [63]. The dysregulation of this pathway has been linked to uncontrolled cell proliferation and tumor formation.

Upon binding to their receptors, Wnt ligands stabilize and translocate β-catenin into the nucleus. In the nucleus, β-Catenin interacts with TCF/LEF transcription factors, promoting gene expression in cell cycle progression (e.g., MYC, Cyclin D1) [64,65]. The overactive β-catenin has been shown to cause an increase in cell proliferation, invasion, and apoptosis resistance, which contributes to the development of odontogenic cancer [66].

Ki-67 is a widely used protein marker for cell proliferation in various tumors, including odontogenic tumors, as it reflects the growth fraction of cells within a tumor. It is expressed during the active phases of the cell cycle (G1, S, G2, and mitosis) but is absent in the resting phase (G0). In odontogenic tumors, Ki-67 expression serves as an indicator of growth potential and aggressiveness. A high Ki-67 labeling index suggests a more rapidly proliferating and potentially aggressive tumor [46,67,68,69]. Carreón-Burciaga et al. demonstrated that Ki-67 expression is significantly higher in AC than in ameloblastomas, correlating with the aggressive nature of AC, including invasive behavior and metastatic potential [70]. However, despite its usefulness, Ki-67 alone does not provide a definitive distinction between benign and malignant tumors.

PITX2, a transcriptional factor, is expressed during the initial phases of morphogenesis and odontogenesis. Its function involves several processes: cell proliferation, migration, invasion, and tumor growth. PITX2 is expressed in various types of tumors, including colon, prostate, ovary, and breast cancers. Garcia-Munoz et al. have demonstrated that in AC, PITX2 is activated through the WNT cellular signaling pathway and that its expression level is associated with the tumor’s aggressiveness [71]. Hii et al. have demonstrated that, in addition to PITX2 activation, the presence of Zinc Finger E-box Binding Homeobox 1 (*ZEB1*) and Meis homeobox 2 (*MEIS2*) genes is observed in AC, while the genes Distal-Less Homeobox 2 (*DLX2*), Distal-Less Homeobox 3 (*DLX3*), and Msh Homeobox 2 (*MSX2*) are absent in clear cell odontogenic carcinoma [72]. ZEB1 is very important in the change from normal cells to cancer cells. This change is called the epithelial-to-mesenchymal transition (EMT). EMT is a key process in the malignant transformation of ameloblastoma. Malignant transformation is the change that causes cancer cells to grow and spread. This process is influenced by TGF-β (Transforming Growth Factor-Beta) signaling [73]. Normally, ZEB1 is a protein that controls which genes are turned on or off. It turns on genes that make cells act like mesenchymal cells, and it turns off genes that make cells act like epithelial cells. This process is called the epithelial-to-mesenchymal transition or EMT. However, when TGF-β is present, ZEB1 levels increase, which causes a downregulation of E-cadherin (a protein that helps cells stick together), an upregulation of N-cadherin and vimentin (markers of mesenchymal cells), and a higher rate of the migration and invasion of ameloblastoma cells, which could potentially lead to malignant transformation [73].

CD147 is a type I transmembrane glycoprotein found mostly on the cell surface, where it functions as a receptor and cell adhesion molecule. It is widely expressed in different tissues and cell types and is well known for its role in cancer progression. It promotes tumor invasion and metastasis by interacting with extracellular matrix proteins and facilitating the secretion of matrix metalloproteinases (MMPs) that degrade the extracellular matrix. CD147 was identified in AC [74]. It has also been shown that in AC, there is very low expression of claudins, a family of integral membrane proteins that play a crucial role in the formation of tight junctions between cells in various tissues. The presence of abnormalities in the formation of these junctions promotes tumor growth. Reduced claudin expression is associated with tight junction disassembly, which can promote tumor invasion and has been observed in oral squamous cell carcinoma [74,75].

Integrins are a family of transmembrane proteins that mediate cell–cell interactions and, together with the extracellular matrix, are critical for various cellular processes, including cell adhesion, signaling, migration, and tissue organization. Integrins a3β1 and a5β1 are highly expressed in ameloblastoma and AC. According to some authors, their upregulation would indicate a more aggressive tumor phenotype, predisposing to local invasion and metastasis [76].

Syndecans are a family of transmembrane heparan sulfate proteoglycans that play essential roles in various cellular processes. Low levels of syndecan-1 expression correlate with tumor progression and development (found in multicystic ameloblastomas and AC). This variation in expression may reflect differences in tumor aggressiveness and behavior [77,78].

Tenascins are a family of extracellular matrix (ECM) glycoproteins that play critical roles in biological processes such as tissue development, wound healing, and immune responses. By interacting with other ECM components, cell surface receptors, and signaling pathways, tenascins influence cell behavior and tissue structure. Particularly, Tenascin-C (TNC) and Tenascin-W (TNW) contribute to tumor progression, metastasis, and resistance to therapy [79]. Tenascins have been observed to interact with cell surface receptors, including integrins and epidermal growth factor receptors (EGFRs), to promote tumor cell proliferation [79,80]. TNC, for instance, has been shown to enhance the activation of growth factor signaling pathways such as EGFR and Wnt/β-catenin, thereby promoting increased tumor cell survival and division [81]. Furthermore, TNC and TNW have been shown to create a permissive environment for cancer cell migration by modulating cell adhesion and promoting epithelial-to-mesenchymal transition (EMT). TNC disrupts normal cell–extracellular matrix (ECM) interactions, allowing cancer cells to detach and invade surrounding tissues [82]. It has been demonstrated that TNC supports metastasis by facilitating tumor cell survival in the bloodstream and aiding colonization at distant sites [81]. TNC has been shown to provide survival signals to cancer cells, thereby rendering them more resistant to chemotherapy and radiotherapy. It also functions as a barrier to drug penetration by modifying the ECM [82]. TNC suppresses immune responses by inhibiting T-cell activation and promoting an immunosuppressive tumor microenvironment. This contributes to resistance against immune checkpoint inhibitors.

Perilipin is a key protein involved in lipid metabolism, primarily regulating fat storage in adipocytes. Increased perilipin-1 and perilipin-2 (also known as adipophilin) expression has been linked to EMT, a key process in odontogenic tumor invasion [83]. EMT allows tumor cells to detach from the primary site, migrate, and invade surrounding bone and soft tissue, which is common in aggressive odontogenic tumors such as ameloblastoma and ACs. Odontogenic tumors often thrive in hypoxic environments, where perilipins help cancer cells adapt by preventing lipid peroxidation and oxidative stress. This contributes to tumor survival, resistance to therapy, and recurrence. A study by Sánchez-Romero et al. identified lipid droplets immunoexpressing perilipin-1 and perilipin-2 (also known as adipophilin) in the cytoplasm of both ameloblastoma and AC cells, with stronger expression observed in AC samples [83].

Research on SOX2 in odontogenic cancer is still emerging. It has been established that SOX2 functions as a critical marker of cancer stem-like cells (CSCs), a specific subpopulation of tumor cells that possess self-renewal capacity and therapy resistance [84]. The presence of CSCs has been identified as a contributing factor to the initiation, progression, and recurrence of aggressive odontogenic tumors [84,85]. High SOX2 expression has been associated with an undifferentiated state, a condition that enables tumor cells to proliferate indefinitely [86].

To date, there are conflicting data in the literature regarding SOX2 expression in AC. According to Silva et al., the higher expression of SOX2 has significant self-renewal and proliferative properties [87]. Instead, Lei et al. found that a strong and diffuse nuclear expression of SOX2 was a specific (86%) and sensitive (77%) marker for AC [61]. This expression was essentially negative in most cases of ameloblastoma. The authors recommended the use of SOX2 in combination with Ki-67 in a panel for the diagnosis of ameloblastic neoplasms [88,89]. However, Robinson et al. found that overall SOX2 expression was higher in ameloblastoma cases than in ACs, demonstrating the limited utility of this marker in distinguishing the two entities [68]. Mishra et al.’s findings indicated that SOX2 was the only marker that significantly discriminated between ameloblastoma and AC (with an RR of −0.19) [90].

Table 3 provides a comprehensive overview of the molecules implicated in the formation and progression of AC.

### 3.6. Genetics

As is well established, the tumor suppressor gene *Tumor Protein p53 (TP53)*, which is located on chromosome 17p13, plays a critical role in regulating cell cycle arrest, apoptosis, and maintaining genomic stability. *TP53* mutations are among the most frequent genetic alterations in human tumors, leading to increased cell proliferation and enhanced malignant potential [91].

In the context of AC, studies have examined the expression of the p53 protein, the product of the *TP53* gene, to understand its involvement in tumor pathogenesis [92,93,94]. For example, a study by Kumamoto et al. observed higher p53 expression in plexiform ameloblastoma cases compared to follicular types, suggesting that alterations in the p53 pathway may contribute to oncogenesis or the malignant transformation of odontogenic epithelium [92]. In addition, research has shown that the hypermethylation of the p16 tumor suppressor gene is present in both ameloblastoma and AC, suggesting that epigenetic alterations, in addition to *TP53* alterations, are involved in the tumorigenesis of these lesions [92,94]. However, findings regarding p53 expression in AC are not entirely consistent. A comparative study analyzing p53 expression in various odontogenic lesions, including ameloblastoma, AC, and other odontogenic lesions, reported significantly lower p53 immunopositivity in AC compared to the other lesions studied [95]. This suggests that while p53 alterations may play a role, other molecular mechanisms may also be critical in the pathogenesis of ameloblastic carcinoma. Collectively, these studies suggest that *TP53* mutations and p53 protein expression are involved in the development and progression of AC.

The *BRAFV600E* mutation is present in a significant proportion of ameloblastomas [96]. In their study, Diniz et al. reported the *BRAFV600E* mutation in approximately 82% of benign ameloblastomas and approximately 38% of ACs [97]. González-González et al. revealed that the BRAF-V600E mutation is related to the aggressive behavior of AC, and early radical resection is crucial [98].

In vitro experiments demonstrated that BRAF-mutated ameloblastoma cells exhibited the constitutive activation of the MAPK pathway, which was inhibited by the BRAF inhibitor vemurafenib [99,100]. At present, Dabrafenib is the most widely used drug in the targeted therapy of ACs. It has been demonstrated to induce a reduction in the size of the tumor, accompanied by mild side effects, including skin problems, dry eyes, changes in voice tone, and systemic symptomatology, such as fatigue, joint pain, and nausea [101].

Recent genomic studies have identified mutations in the gene *Smoothened (SMO)*, which encodes the smoothened receptor, as a major contributor to the pathogenesis of these tumors [102,103]. Mutations in SMO have been identified in approximately 10.6% to 39% of ameloblastoma cases. The most commonly identified mutations include p.Leu412Phe (L412F), p.Trp535Leu (W535L), and p.Gly416Glu (G416E) [102]. These mutations lead to the constitutive activation of the Hedgehog signaling pathway, promoting uncontrolled cell proliferation and tumor growth [103].

Notably, SMO mutations show a distinct predilection for tumors located in the maxilla. Studies have reported that a significant proportion of maxillary ameloblastomas harbor SMO mutations [102], whereas mandibular tumors are more likely to harbor BRAF mutations [104]. This anatomical correlation suggests different molecular mechanisms underlying tumor development, depending on the site.

### 3.7. Differential Diagnosis

Immunohistochemistry allows AC to be distinguished from all those neoplastic lesions that mimic its histological and clinical features, such as primary intra-alveolar epidermoid carcinoma, squamous cell carcinoma arising from the lining of an odontogenic cyst, acanthomatous ameloblastoma, kerato-ameloblastoma, mandibular odontogenic myxoma, squamous odontogenic tumor, calcifying odontogenic tumor, salivary gland neoplasms such as a pseudo-adamantine tumor, and angiosarcoma of the jaw, angiosarcoma of the jaw, mandibular odontogenic myxoma, odontogenic squamous cell tumor, odontogenic calcifying epithelial tumor, salivary gland neoplasms such as pseudo-adamantine adenocarcinoma, ductal carcinoma, high-grade mucoepidermoid carcinoma and metastatic carcinoma to the jaw from the lung, breast and gastrointestinal tract [105,106]. Finally, it is necessary to exclude that the mass is not due to bone metastasis or the invasion of the adjacent soft tissues or sinuses by a tumor.

### 3.8. Clinical Course and Treatment

The clinical course is typically aggressive, with extensive locoregional invasion and distant metastases [22]. This appears to be the most important prognostic factor. The first symptoms are swelling, pain, and numbness; less common symptoms are paresthesia (especially hypoesthesia) or anesthesia, tooth loss, etc. [13,107]. The path of diffusion is both hematogenous and lymphatic [108]. From the literature, the lung is the most common site of metastasis, both in the maxilla and the mandible [107,108]:

- In total, 29.41% of AC arising from mandible determines distant metastases and precisely lung 54.54%, lymph nodes 38.2%, bone 18.18%, pleura 11%, liver 7.2%, skull base 7.2%, brain 3.6%, kidney 3.6%, disease relapse 1.8%, parotid 1.8%, spleen 1.8%, diaphragm 1.8%, ilium 1.8%, mediastinum 1.8%, and extensive metastatic disease 34.54%.
- Overall, 25.97% of AC arising from maxilla determines distant metastases and precisely lung 40%, lymph nodes 30%, bone 20%, skull base 10%, disease relapse 10%, brain 5%, orbit 5%, pterygomaxillary fossa 5%, mandible 5%, small intestine 5%, and extensive metastatic disease 25%.
- In total, 33.33% of AC arising from the skull base determines intracranial metastases.

Our analysis shows that although the skull base is a rare site (only 1.04%), it is the most frightening site compared to the mandible and maxilla; it metastasizes in 33.33% of cases (compared to 29.42% and 25.97% of cases for the mandible and maxilla, respectively).

There is no standard treatment, and many relapses occur [81]. Long follow-up is recommended. Wide local excision appears to be the treatment of choice, as concluded by most investigators [55]. Some authors support bone margins of 2–3 cm for mandibular AC and 1–1.5 cm for maxillary AC with an average of one block removal [23,35,109]. Cervical lymph node dissection should only be considered if there is evidence of lymphadenopathy. Surgical intervention frequently leads to the formation of complex bone defects, which pose significant challenges in terms of management due to their anatomical, functional, and esthetic complexity. In light of these challenges, some authors have proposed the use of an osteosynthesis plate to restore the continuity of the mandibular arch and preserve its functionality [25]. However, this therapeutic option is associated with esthetic impairment. Free flap reconstruction has become the preferred method due to its versatility and reliability. The selection of a flap is contingent upon the dimensions of the defect, its localization, and the overall health of the patient. The free flap is frequently utilized for mandibular reconstruction, offering a substantial length of bone that is conducive to dental rehabilitation [12,37]. Additionally, it can be harvested with a skin paddle for soft tissue reconstruction. Conversely, the anterolateral thigh (ALT) free flap is predominantly utilized for soft tissue reconstruction. It can be used to provide soft tissue support for osteosynthesis plaques [109].

In a recent study, Ismail et al. proposed the use of a vascularized composite graft as an alternative treatment for complex jaw defects. This procedure was performed on a 39-year-old patient who underwent hemimaxilectomy for CA and had been disease-free for five years. The defect was managed in a two-step approach. Initially, a composite graft was processed ectopically using autogenic cells of the stromal vascular fraction seeded on an allogeneic devitalized bone matrix. The resultant graft was then loaded with bone morphogenic protein-2 (BMP-2), wrapped within the large dorsal muscle, and pediculated with an arteriovenous bundle. Subsequently, the prefabricated graft was transferred to the site of the defect using an orthotopic technique, and revascularization was achieved through the implementation of microvascular surgical techniques. The graft contained vascularized bone tissue embedded in muscle tissue. Ismail et al. attest to the reliability of the technique for a follow-up period of two years, and they report a lower morbidity of the donor site and an improvement in oral rehabilitation at the expense of slight bone resorption [20].

The effectiveness of adjuvant treatment (radiotherapy and/or chemotherapy) is not yet clear. So far, there is not enough evidence showing a real benefit in these adjuvant treatments. However, they are the only option for locally advanced or metastatic disease that is not amenable to surgical resection. The efficacy of chemotherapy in patients with metastatic AC has been studied for years with no significant response or survival benefit. To date, there are no clear opinions [38,110].

The role of radiotherapy in the management of this tumor remains a subject of debate. In cases where complete surgical excision is challenging or when there is advanced soft tissue invasion, postoperative radiotherapy is often considered [111]. Li et al. underscored the importance of wide local excision, followed by postoperative RT, to mitigate the risk of recurrence [43]. In a separate study, Takahashi et al. proposed a single-fraction helical tomotherapy approach for the management of residual AC following surgical resection [112]. For patients deemed unsuitable candidates for surgery due to the presence of medical comorbidities or issues with tumor resectability, radiotherapy has been utilized as a definitive treatment modality. Aoki et al. reported positive outcomes in an elderly male with poor general health and maxillary AC, treated exclusively with radiotherapy. A decade after the treatment, no major adverse event, complication from irradiation, local recurrence, or metastasis was observed [11].

A systematic review and individual patient data analysis of 199 AC cases showed that achieving clear surgical margins (R0 resection) is associated with favorable survival outcomes. The study found no significant survival benefit from adjuvant RT; however, older patients with high-risk factors may benefit from postoperative radiation [55].

The possible beneficial role of carbon ion therapy has been demonstrated by Jensen et al. [113]. Proton beam therapy (PBT) has been explored as a treatment modality for AC, particularly in cases where surgery is not feasible. PBT has the advantage of delivering high doses of radiation with precision, minimizing damage to surrounding healthy tissue [14,18].

The identification of SMO mutations in AC has therapeutic implications. Preclinical studies have shown that tumors with SMO p.Leu412Phe mutations respond to inhibitors of the Hedgehog pathway, such as arsenic trioxide (ATO). ATO, an FDA-approved drug for acute promyelocytic leukemia, has shown the potential to inhibit the oncogenic effects of SMO mutations in ameloblastoma cells [101].

A recent study by Oh et al. showed a significant expression of PD-L1 (compared to other malignant dental tumors analyzed), suggesting the involvement of adaptive immune resistance [114]. This supports the therapeutic potential of immune checkpoint inhibitors in patients with these rare malignancies [114,115].

### 3.9. Prognosis

The lack of standard treatment protocols affects prognosis. According to the extant literature, a recurrence can occur within a broad timeframe.

Deng et al. [17] showed that the type of treatment does not significantly influence survival. Uzawa et al. [116] showed that prophylactic neck dissection does not increase overall survival. Postoperative radiotherapy is effective in older patients with risk factors but does not affect overall survival in young patients with localized disease [117].

## 4. Discussion

AC is a rare but significant odontogenic neoplasm that constitutes less than 2% of all odontogenic tumors but is responsible for approximately 30% of malignant neoplasms in this category. Demographic analysis reveals a higher prevalence in men, with a male-to-female ratio of approximately 2.45:1. The mandible is the most commonly affected site, accounting for approximately 66% of cases, with a mandible-to-maxilla ratio of 2:1. The mean age of onset is around 46.93 years, which is consistent with the tendency for AC to manifest at a later stage in life compared to other odontogenic tumors. The substantial variation in age and sex ratios across different studies indicates the potential influence of regional or population-based factors on the presentation of these cancers. A comprehensive review of 126 studies revealed that while AC accounts for less than 2% of all odontogenic tumors, it accounts for approximately 30% of malignant odontogenic tumors. This finding underscores the necessity of acknowledging AC as a notable clinical entity despite its infrequency [56].

AC is distinguished by its aggressive clinical course, which is characterized by extensive locoregional invasion and considerable potential for distant metastases [22,107,108]. The extent of invasion and metastatic spread is widely regarded as the most critical prognostic factor influencing patient outcomes [17,55,56,90,98,101]. The initial symptoms of AC often include progressive swelling, pain, tooth loss, and numbness in the affected region. In some cases, patients may also manifest tooth mobility and experience paresthesia, particularly hypoesthesia or anesthesia, which may signify perineural invasion. Rare manifestations may encompass the ulceration of the overlying mucosa and trismus in cases where the tumor extends into the masticatory space [67,85,90,95].

AC manifests a dual mode of dissemination: hematogenous and lymphatic spread. The tumor frequently metastasizes to cervical lymph nodes, necessitating a careful evaluation of regional lymph nodes during staging and treatment planning. Moreover, distant organs are often affected, with the lungs being the most common site of metastasis, followed by the liver and bones [6,17,55,56]. The growth of these metastases is rapid and reflects the extremely malignant nature of AC compared to other odontogenic tumors. These characteristics are associated with distinct molecular profiles; however, further research is necessary to elucidate the underlying mechanisms. The retrospective nature of the included studies, the incompleteness of the medical records, and the limited follow-up periods likely contributed to an underestimation of the actual survival rate. In contrast to benign ameloblastomas, which demonstrate a lower recurrence rate, ACs exhibit a relatively high recurrence rate ranging from 40% to 60% [56,91,108]. This recurrence manifests after a median period of 12 months, with a marked escalation in probability as time elapses. The presence of recurrence and metastasis, particularly in advanced stages, is often associated with a poor prognosis, underscoring the significance of early and aggressive intervention.

Distinguishing between AC and ameloblastoma can be challenging, especially when cytologic atypia is minimal, according to histopathological analysis. The differential expression of immunohistochemical markers, such as SOX2, tenascins, and PITX2, has been observed between these tumors, suggesting potential diagnostic and prognostic value.

Research has demonstrated that AC displays a diffuse and strong nuclear staining pattern for SOX2, indicative of a high-grade malignancy. This pronounced expression suggests that SOX2 may serve as a potential marker for identifying malignant transformation in ameloblastic neoplasms [61]. Conversely, ameloblastomas exhibit reduced SOX2 expression, predominantly restricted to peripheral ameloblast-like cells, accompanied by diminished staining intensity [118].

PITX2 expression has been shown to be significantly elevated in AC compared to ameloblastomas in other studies. This heightened expression may be associated with the aggressive behavior and malignant transformation observed in ameloblastoma [71].

The differential expression patterns of SOX2 and PITX2 underscore their potential diagnostic and prognostic value in distinguishing AC from ameloblastoma. SOX2’s association with cancer stem cell behavior and PITX2’s involvement in the WNT signaling pathway underscore their roles in the pathogenesis and progression of these tumors [61,71]. Consequently, the incorporation of these markers into standard immunohistochemical panels could potentially enhance diagnostic precision and inform treatment strategies.

While the extant literature provides valuable insights, it is not without limitations. Most reviewed studies are retrospective, and numerous cases exhibit inadequate documentation and brief follow-up periods. These limitations can lead to biased estimates of survival rates and unreliable predictions of recurrence. Additionally, the dearth of large-scale, multicenter studies necessitates the use of small case series, which are often constrained by geographical biases. For instance, Asia has the highest proportion of AC cases, which may not be reflective of global patterns.

The complexity of these tumors necessitates further investigation to establish definitive molecular markers and treatment protocols. The molecular pathogenesis of AC remains incompletely understood, and while targeted therapies have shown promise in other oncologic contexts, the application of such therapies to AC has yet to be fully explored. The elucidation of mutations and alterations in signaling pathways has the potential to unveil novel therapeutic targets, offering a glimmer of hope for patients afflicted with recurrent or metastatic AC. Concomitantly, surgical resection remains the cornerstone of treatment; however, the use of adjuvant therapies such as chemotherapy or radiotherapy is limited, given AC’s radioresistant nature.

## 5. Conclusions

In conclusion, AC, despite its rarity, poses considerable diagnostic and therapeutic challenges in the domain of odontogenic tumors. Its aggressive behavior, potential for metastasis, and complex histopathological features underscores the necessity for ongoing research into molecular markers, prognostic factors, and effective treatment strategies. Enhanced documentation and prolonged follow-up periods are imperative to refine our understanding of these tumors and enhance patient outcomes. This article aims to deepen our understanding of this complex disease. The advancement of clinical, biological, and etiopathological knowledge may facilitate earlier diagnosis and treatment, leading to substantial improvements in patients’ quality of life and long-term survival.

## Figures and Tables

**Table 1:** Clinical characteristics of patients with follow-ups of more than 5 years.

No.	Year	Author	Ref.	Age	Sex	Site of Involvement	Metastases
1	1984	Slootweg and Muller et al.	[4]	23	F	Mandible	-
2	2004	Goldenberg et al.	[7]	60	F	Mandible	Yes (brain)
3	2016	Fonseca et al.	[8]	27	F	Mandible	-
4	2016	Loyola et al.	[9]	38	M	Mandible	-
5	2016	Loyola et al.	[9]	36	M	Mandible	-
6	2016	Loyola et al.	[9]	60	M	Mandible	-
7	2016	Fomete et al.	[10]	55	M	Maxilla	-
8	2016	Fomete et al.	[10]	32	M	Maxilla	-
9	2018	Aoki T. et al.	[11]	80	M	Maxilla	Yes (lymph nodes)
10	2018	Fahradyan A. et al.	[12]	15	M	Mandible	-
11	2018	Soyele O.O et al.	[13]	44	M	Maxilla	-
12	2018	Soyele O.O et al.	[13]	54	M	Mandible	-
13	2018	Soyele O.O et al.	[13]	47	M	Mandible	-
14	2018	Soyele O.O et al.	[13]	32	M	Mandible	-
15	2018	Soyele O.O et al.	[13]	46	M	Mandible	-
16	2018	Soyele O.O et al.	[13]	36	F	Mandible	-
17	2018	Yamagata K. et al.	[14]	70	F	Maxilla	Yes (pterygomaxillary fossa)
18	2019	Kosanwat T. et al.	[15]	46	F	Maxilla	Yes (left orbit)
19	2019	Landeen K. et al.	[16]	27	F	Maxilla	Yes (skull base)
20	2019	Deng L. et al.	[17]	36	M	Mandible	-
21	2019	Deng L. et al.	[17]	40	F	Mandible	-
22	2019	Deng L. et al.	[17]	47	M	Maxilla	-
23	2019	Deng L. et al.	[17]	61	M	Mandible	-
24	2019	Deng L. et al.	[17]	40	M	Mandible	-
25	2019	Deng L. et al.	[17]	39	F	Mandible	-
26	2019	Deng L. et al.	[17]	42	M	Mandible	-
27	2019	Deng L. et al.	[17]	46	M	Mandible	-
28	2019	Deng L. et al.	[17]	32	M	Mandible	-
29	2020	Takayama K. et al.	[18]	71	M	Maxilla	-
30	2020	Cho et al.	[19]	45	M	Mandible	-
31	2021	Ismail et al.	[20]	39	F	Maxilla	-
32	2022	Hoehnle et al.	[21]	31	M	Maxilla	Local disease progression
33	2023	Chen et al.	[22]	68	M	Maxilla	-
34	2023	Ogane et al.	[23]	72	F	Mandible	-
35	2023	Hurnik et al.	[24]	-	M	Mandible	Yes (lymph nodes, orbit, lungs)
36	2023	Barca et al.	[25]	33	M	Mandible	Yes (lymph nodes, infratemporal fossa, orbit)

**Table 2:** Clinical characteristics of patients with follow-ups of less than 5 years.

No.	Year	Author	Ref.	Age	Sex	Site of Involvement	Metastases
1	1984	Slootweg and Muller et al.	[4]	75	M	Mandible	-
2	1988	Dorner et al.	[26]	84	M	Mandible	Yes (lung)
3	1991	Bruce and Jackson et al.	[27]	57	M	Mandible	Yes (lung)
4	1998	Simko et al.	[28]	64	F	Mandible	Yes (lung)
5	1998	Infante-Cossio et al.	[29]	69	F	Maxilla	-
6	1998	Infante-Cossio et al.	[29]	77	M	Maxilla	Yes (brain)
7	1998	Infante-Cossio et al.	[29]	64	M	Maxilla	-
8	2003	Kawauchi S et al.	[30]	67	M	Mandible	-
9	2003	Datta et al.	[31]	22	M	Mandible	Yes (bone)
10	2003	Oginni et al.	[32]	65	M	Mandible	-
11	2007	Hall et al.	[33]	27	M	Mandible	-
12	2007	Hall et al.	[33]	43	F	Mandible	Yes (lung, liver)
13	2007	Hall et al.	[33]	50	M	Mandible	-
14	2007	Hall et al.	[33]	49	M	Mandible	-
15	2007	Hall et al.	[33]	53	F	Mandible	-
16	2007	Hall et al.	[33]	59	M	Mandible	-
17	2007	Hall et al.	[33]	75	M	Maxilla	-
18	2007	Hall et al.	[33]	17	F	Mandible	-
19	2007	Hall et al.	[33]	63	M	Maxilla	-
20	2007	Hall et al.	[33]	31	M	Mandible	-
21	2007	Hall et al.	[33]	15	M	Maxilla	-
22	2007	Hall et al.	[33]	16	M	Maxilla	Yes (lymph nodes)
23	2007	Hall et al.	[33]	7	F	Maxilla	-
24	2007	Hall et al.	[33]	52	M	Maxilla	-
25	2007	Benlyazid et al.	[34]	90	M	Maxilla	-
26	2010	Ram et al.	[35]	21	M	Mandible	-
27	2010	Lucca et al.	[36]	73	M	Maxilla	-
28	2010	Lucca et al.	[36]	69	M	Maxilla	-
29	2010	Jeremic et al.	[37]	58	M	Mandible	Yes (lymph nodes, lung)
30	2012	Horvath et al.	[38]	8	F	Mandible	Yes (lung, bone)
31	2012	Pirklbauer et al.	[39]	86	M	Mandible	Yes (lung, skull)
32	2012	Kamath et al.	[40]	75	M	Mandible	-
33	2013	Yoshioka et al.	[41]	17	M	Mandible	Yes (lung, skull)
34	2014	Kar et al.	[42]	70	M	Mandible	Yes (lymph nodes)
35	2014	Li et al.	[43]	75	M	Mandible	Yes (lung)
36	2014	Nobusawa et al.	[44]	84	F	Maxilla	Yes (liver)
37	2016	Loyola et al.	[8]	21	M	Mandible	-
38	2018	Kikuta S. et al.	[45]	62	M	Mandible	Yes (lungs, liver kidney)
39	2018	Mahmoud M.A.S et al.	[46]	29	F	Mandible	-
40	2018	Mahmoud M.A.S et al.	[46]	62	M	Maxilla	-
41	2018	Soyele O.O et al.	[13]	34	M	Mandible	-
42	2018	Soyele O.O et al.	[13]	5	F	Maxilla	-
43	2018	Soyele O.O et al.	[13]	49	M	Mandible	-
44	2018	Soyele O.O et al.	[13]	60	F	Maxilla	-
45	2018	Soyele O.O et al.	[13]	32	M	Mandible	-
46	2018	Soyele O.O et al.	[13]	24	F	Mandible	-
47	2018	Soyele O.O et al.	[13]	16	F	Mandible	-
48	2019	Deng L. et al.	[17]	30	M	Mandible	-
49	2019	Deng L. et al.	[17]	35	M	Mandible	-
50	2019	Deng L. et al.	[17]	75	M	Mandible	Yes (lung)
51	2019	Deng L. et al.	[17]	57	M	Maxilla	-
52	2019	Deng L. et al.	[17]	25	F	Mandible	-
53	2019	Deng L. et al.	[17]	62	F	Maxilla	Yes (lymph nodes)
54	2019	Deng L. et al.	[17]	48	M	Mandible	-
55	2019	Deng L. et al.	[17]	72	M	Maxilla	Yes (lymph nodes)
56	2019	Deng L. et al.	[17]	54	M	Mandible	-
57	2019	Sancheti S. et al.	[47]	31	M	Mandible	-
58	2020	Yukimori A. et al.	[48]	26	M	Mandible	--
59	2020	Tarle M. et al.	[49]	64	M	Maxilla	Yes (brain)
60	2020	Vu et al.	[50]	2	F	Mandible	-
61	2021	Behtaj et al.	[51]	20	F	-	-
62	2022	Manchanda et al.	[52]	55	M	Mandible	-
63	2023	Schuch et al.	[53]	23	F	Maxilla	-
64	2023	Harada et al.	[54]	76	M	Mandible	Spine

**Table 3:** Molecular mechanisms and pathophysiology of AC.

Name	Molecular Mechanisms	Pathophysiology
Wnt/β-Catenin pathway	Cell proliferation, differentiation, and survival, and epithelial–mesenchymal interactions.	Cell proliferation, invasion, and apoptosis resistance.
Ki-67	Cell proliferation.	Cell proliferation and invasion
PITX2	Cell proliferation, differentiation, and survival.	Cell proliferation, migration, invasion, and tumor growth.
CD147	Transmembrane receptor involved in cell adhesion.	Cancer progression and promotes tumor invasion and metastasis.
Integrins a3β1 and a5β1	Mediate cell–cell interactions, together with the extracellular matrix, are critical for cell adhesion, signaling, migration, and tissue organization.	Local invasion and metastasis.
syndecan-1	Cell proliferation, differentiation, and survival.	Tumor progression and development.
Tenascin-C and Tenascin-W	Tissue development, wound healing, and immune responses.	Tumor progression, invasion, and extracellular matrix remodeling, metastasis, and resistance to therapy.
Perilipin	Lipid metabolism, primarily regulating fat storage in adipocytes	Invasion of surrounding bone and soft tissues.
SOX2	Tooth development.	Aggressive tumor behavior, recurrence, and a poor prognosis.

## Data Availability

The data are available on request from the corresponding author.
